# Prise en charge du syndrome alport au cours de la grossesse: à propos d’un cas et revue de la litterature

**DOI:** 10.11604/pamj.2018.31.241.12581

**Published:** 2018-12-20

**Authors:** Taher Berrada, Oumaima M’hamdi, Intissar Benzina, Fatema Zahra Lamine, Najia Zraidi, Abdelaziz Baidada

**Affiliations:** 1 Service de Gynécologie-Obstétrique et Endoscopie Gynécologique M1 Maternité Souissi, Rabat, Maroc; 2 Service de Gynécologie-Obstétrique et Endocrinologie Gynécologique M3 Maternité Souissi Rabat, Maroc

**Keywords:** Syndrome d´alport, grossesse, insuffisance rénale, Alport syndrome, pregnancy, renal failure

## Abstract

La survenue d'une grossesse chez une patiente porteuse d'un syndrome d'alport est une situation rare vu les répercussions de cette néphropathie sur la fertilité. Nous rapportons un cas de syndrome d'alport associé à la grossesse chez une parturiente âgée de 28 ans. La survenue d'une menace d'accouchement prématuré avec une élévation des chiffres tensionnels a nécessité l'hospitalisation de cette femme pour une éventuelle prise en charge. Une revue de la littérature discutant les différentes complications materno-fœtales et s'intéressant á la prise en charge au cours de la grossesse est également présentée.

## Abstract (en)

The occurrence of pregnancy in a patient with alport syndrome is rare because this renal disease can impact fertility. We report a case of alport syndrome associated with pregnancy in a parturient woman aged 28 years. Possible premature delivery associated with elevated blood pressure led to hospitalization for patient's mangement. We here report a literature review on the various maternal-foetal complications of alport syndrome and we focus on the management of alport syndrome during pregnancy.

## Introduction

La grossesse chez les femmes atteintes d'une insuffisance rénale chronique au stade de dialyse est rare, sa prise en charge est accompagnée d'un certain nombre de complications maternelles et fœtales nécessitant une collaboration entre néphrologues et gynécologue-obstétriciens [1]. Nous rapportons un cas d'une parturiente suivie pour un syndrome d'Alport au stade d'insuffisance rénale terminale prise en charge à la maternité Souissi de Rabat. Le but de notre travail est d'éclaircir la prise en charge du syndrome d'alport au cours de la grossesse à partir de cette observation et d'une revue de la littérature.

## Patient et observation

Il s'agit de Madame Y.A âgée de 28 ans. 4^ème^ gestes 2^ème^ pare, un enfant vivant. Le 1^er^ geste était un accouchement par voie basse d'un nouveau-né à terme de sexe masculin, poids de naissance 3200g, avec un bon développement psychomoteur et staturo-pondérale. Le 2^ème^ et 3^ème^ geste étaient des avortements à 2 mois non curetés. Le 4^ème^ geste est la grossesse actuelle estimée à 8 mois. Notre patiente ayant comme antécédent une hypoacousie depuis l'enfance. Suivie depuis sept ans pour une hypertension artérielle sous amlodipine, qui s'est rapidement aggravée par une insuffisance rénale terminale nécessitant trois séances d'hémodialyse chaque semaine. Une ponction biopsie rénale a été réalisée et le syndrome d'alport a été confirmé par étude histologique. Durant la grossesse actuelle la patiente a été mise sous alpha-méthyldopa 500mg toutes les 8 heures pour le traitement de l'hypertension artérielle avec 3 séances d'hémodialyse hebdomadaire. La patiente a été admise au service des expectantes de la maternité Soussi de Rabat pour menace d'accouchement prématurée, avec des chiffres tensionnels élevés. L'examen à l'admission trouve une patiente consciente, bien orientée dans le temps et dans l'espace, un poids à 50kg, une taille de 1m57, une température à 37°C avec une anamnèse infectieuse négative, une tension artérielle à 18/10 cmHg sans signes neurosensoriels. Sur le plan obstétrical, la patiente avait une hauteur utérine à 23cm avec des contractions utérines présentes, les bruits cardiaques fœtaux étaient perçus au stéthoscope de Pinard. Le toucher vaginal a trouvé un col mi-souple en voie de centralisation, effacé à 60% dilaté à 2 doigts, une présentation céphalique mobile avec une poche des eaux intacte. L'échographie obstétricale avait objectivé une grossesse monofœtale évolutive, avec activité cardiaque positive, une présentation céphalique, un liquide amniotique en quantité normal, un diamètre bipariétal à 78mm et une longueur fémorale à 60 mm. Ce qui correspond à des mensurations de 31 SA+4j, avec un doppler obstétrical normal. La mesure de la longueur du col était à 18mm. L'étude du rythme cardiaque fœtal a mis en évidence un tracé oscillant et réactif avec un rythme de base à 140 battements par minute. La patiente a été mise sous un tocolytique: un inhibiteur calcique nifédipine, avec un arrêt de l'alpha-méthyldopa, une corticothérapie à base de béthamétasone pour la maturation pulmonaire fœtale à une dose de 12mg, à renouveler après 24h. Le bilan infectieux était négatif. Le bilan sanguin, réalisé, a retrouvé une insuffisance rénale avec taux d'urée à 0.98 g/l et une créatinine à 71.1 mg/l, une kaliémie à 5.58 mEq/l, des réserves alcalines à 19 mEq/l, une anémie à 7.7g/dl, un taux de plaquettes à 211 000/mm^3^. Le bilan hépatique était normal. La patiente a bénéficié de deux séances d'hémodialyse à deux jours d'intervalle sur un circuit hépariné: à l'héparine sodique et l'héparine non fractionnée, sur une durée de 4heures avec une quantité de 2,5 litres. En outre, au cours de la 2^ème^ séance, la patiente a reçu une transfusion en per-dialyse. Au 3^ème^jour de son hospitalisation, malgré la tocolyse, la patiente a accouché d'un prématuré de sexe masculin avec un poids de naissance de 1450g. Le nouveau-né a été transféré en réanimation néonatal pour détresse respiratoire. En post partum direct la parturiente n'a pas présenté de complications, les suites de couches étaient simples.

## Discussion

Le syndrome d'Alport est une affection héréditaire caractérisée par l'association d'une néphropathie hématurique progressive avec des anomalies ultra structurales et immunohistochimiques des membranes basales glomérulaires, d'une surdité de perception d'évolution également progressive et parfois d'anomalies oculaires. Son incidence est de 1/5000 individus. Le syndrome d'Alport est cliniquement et génétiquement hétérogène, le mode de transmission le plus fréquent étant le mode dominant lié à l'X. Il est secondaire à une anomalie de structure du collagène IV, principal constituant des lames basales [2]. Sur le plan clinique, les signes rénaux témoignent d'une atteinte glomérulaire [3]. Ils sont souvent révélateurs, qu'il s'agisse d'une hématurie macroscopique ou d'une protéinurie découverte à l'occasion d'un examen systématique des urines. Chez la femme, la maladie peut être longtemps méconnue et découverte à l'occasion d'une grossesse ou d'une enquête génétique. Le risque de développer une insuffisance rénale terminale avant l'âge de 40 ans est de 12% chez les femmes [4]. L'hypertension artérielle est en général un symptôme tardif. Les signes extra-rénaux sont à type de surdité ou d'hypoacousie parfois latente, découverte par audiogramme. Les atteintes oculaires sont moins fréquentes et concernent le cristallin, la rétine où elles sont spécifiques, et la cornée. Notre patiente avait une hypoacousie dès son enfance, avec apparition d'une hypertension artérielle à l'âge de 21 ans, avec une insuffisance rénale terminale. La biopsie rénale confirme le diagnostic. Le traitement est basé sur la prise des antihypertenseurs afin de ralentir l'aggravation de la protéinurie et le recours à l'hémodialyse en cas d'insuffisance rénale terminale [2]. Pour la plupart des néphropathies, si le débit de filtration glomérulaire est normal au moment de la conception, la grossesse n'entraînera pas d'altération à long terme de la fonction rénale. Le pronostic rénal pendant et dans les suites d'une grossesse est surtout déterminé par le stade de l'insuffisance rénale chronique (valeurs de la créatinine sérique et du débit de filtration glomérulaire durant le premier trimestre), par la sévérité de l'hypertension artérielle et le degré de protéinurie, plutôt que par le type de la maladie rénale préexistante [5]. Sur le plan obstétrical, les parturientes porteuses du syndrome d'alport et d'autres néphropathies ont le risque d'avoir des fausses couches spontanées, d'accouchements prématurés, d'hydramnios, de retard de croissance intra-utérin ainsi qu'un certain nombre d'hématome rétro-placentaire et de mort fœtal in utéro ont été décrit due à l'hypertension artérielle [5]. Notre patiente rapporte la notion de deux avortements non cureté, et elle a été hospitalisé pour une menace d'accouchement prématuré. Les complications maternelles rencontrées sont la prééclampsie et le décès maternel [1]. Au cours de la grossesse, la femme porteuse du syndrome d'alport avec insuffisance rénale terminale, nécessite une prise en charge multidisciplinaire impliquant les néphrologues et les gynécologues obstétriciens. Il est également important que les patientes connues pour des maladies rénales soient informées des risques encourus et puissent planifier la conception et bénéficier, si nécessaire, d'une modification ou d'un ajustement thérapeutique.

Le suivi de leur grossesse doit être plus intensif et rigoureux ainsi: le contrôle optimal des chiffres tensionnels dès la période de conception: éviction des inhibiteurs de l'enzyme de conversion et des diurétiques, ainsi que tout médicament tératogène pour la grossesse, l'alpha-methyldopa et les bêtabloqueurs sont les traitements de référence. La pression artérielle diastolique ciblée entre 80 et 90 mmHg. Au début de la grossesse notre patiente a été mise sous alfa-méthyl-dopa pour la correction de sa tension artérielle. Les inhibiteurs calciques (nifédipine, nicardipine) sont également largement utilisés avec un effet tocolytique [1]. C'était le cas de notre patiente, on a arrêté l'alpha méthyl dopa, qui a été remplacé par la nifédipine pour un double intérêt: tocolytique et anti-hypertensif; 1) le recours à la maturation pulmonaire fœtale par la bethametasone est nécessaire en cas de menace d'accouchement prématuré [6]; 2) l'utilisation de l'aspirine en début de grossesse pour éviter la prééclampsie reste discutée [1]; 3) la dose dialyse doit être augmentée au cours de la grossesse dont le but est d'améliorer le pronostic fœtal. L'utilisation de l'héparine non fractionnée et l'héparine de bas poids moléculaire au cours de la dialyse ne comporte pas de risque hémorragique pour l'accouchement, car il n'y a pas de passage transplacentaire [5]; 4) la prévention ou correction de l'anémie est basée sur la supplémentation martiale et en acide folique (5 mg/j); 5) le traitement par érythropoïétine recombinante si l'hémoglobine est < 9 g/dl. La transfusion en per-dialyse des parturientes n'est pas contre-indiquée, mais elle doit être limitée pour éviter le risque d'immunisation en attendant une transplantation rénale [1]; 6) la prévention de l'acidose métabolique et de l'hypocalcémie et de veiller à un apport protéique et calorique adéquat (apport protéique 1 g/kg/j en cas d'insuffisance rénale); 7) la surveillance de la tension artérielle, de la créatininémie, de l'urée sanguine et de l'uricémie doit être régulière permet l'institution de la dialyse de suppléance est nécessaire si la créatininémie excède 400 µmol/l ou si l'urée sanguine excède 20 mmol/l; 8) l'hospitalisation de la patiente en milieu obstétrical doit être envisagée en cas de majoration de l'hypertension artérielle ou de contractions prématurées. La surveillance régulière de l'état fœtale est fondamentale chez les patientes atteintes de maladie rénale en raison du risque accru de retard de croissance intra-utérin. L'enregistrement des ondes artérielles utérines, entre la 2^e^et la 24^e^ semaine de gestation, est utile pour la prédiction du risque de préclampsie et de retard de croissance intra-utérin. La surveillance fœtale par échodoppler doit être régulière dès le terme de viabilité (à partir de la 26^e^ semaine gestationnelle). Si un retard de croissance intra-utérin est décelé, des évaluations répétées de l'état fœtale, comprenant la cardiotocographie, l'index amniotique et l'enregistrement doppler de l'artère ombilicale et des artères cérébrales du fœtus, aident à reconnaitre une souffrance fœtale et à décider ou non de l'extraction fœtale [7]. En post partum, la prise en continue d'une pilule microprogestative est le moyen contraceptif de choix [1]. Lorsque le diagnostic du syndrome d'alport est porté chez un patient, une enquête familiale précise s'impose, aboutissant à la constitution d'un arbre généalogique. Son intérêt essentiel est, d'une part, de préciser le mode de transmission de la maladie, d'autre part, d'identifier les transmetrices asymptomatiques dans les familles où la maladie est transmise par le chromosome X. Il faut cependant, savoir qu'un examen d'urine négatif n'élimine pas le diagnostic chez les jeunes enfants et que l'hématurie peut rester isolée et intermittente chez la fille transmettrice. ans les familles étudiées et informatives, il est maintenant possible, en se basant sur les études de liaison génétique, de faire le diagnostic génétique précoce et éventuellement prénatal de la maladie. Les renseignements ainsi obtenus sont essentiels pour aborder le conseil génétique et pour discuter éventuellement du don d'organe [2].

## Conclusion

Il est impératif que les patientes suivies pour un syndrome d'alport soient informées des risques encourus et puissent planifier la conception et bénéficier, si nécessaire, d'une modification ou d'un ajustement thérapeutique, afin de mener leur grossesse à terme et d'éviter les complications.

## Conflits d’intérêts

Les auteurs ne déclarent aucun conflit d'intérêts.

## References

[1] Marine Panaye, Anne Jolivot Sandrine Lemoine, Fitsum Guebre-Egziabher, Muriel Doret, Emmanuel Morelon, Laurent Juillard (2014). Pregnancies in hemodialysis and in patients with end-stage chronic kidney disease: epidemiology, management and prognosis. Néphrologie & Thérapeutique.

[2] Marie-Claire Gubler, Laurence Heidet, Corinne Antignac (2007). Syndrome d'alport ou néphropathie héréditaire hématurique progressive avec surdité. Néphrologie & Thérapeutique.

[3] Gubler MC, Heidet L, Antignac C, Jennette JC, Olson JL, Schwartz MM, Silva FG (2007). Alport's syndrome, thin basement membrane nephropathy, nail patella syndrome and type III collagen glomerulopathy. Heptinstall's Pathology of the Kidney.

[4] Jais JP, Knebelmann B, Giatras I, De Marchi M, Rizzoni G, Renieri A (2003). X-linked Alport syndrome: natural history and genotype-phenotype correlations in girls and women belonging to 195 families: a « European Community Alport's syndrome Concerted Action Study ». J Am Soc Nephrol.

[5] Bar J, Ben-Rafael Z, Padoa A (2000). Prediction of pregnancy outcome in subgroups of women with renal disease. Clin Nephrol.

[6] Weyricha Joy, Settera B Anna, Müllerc Alexander, Schmidtc Georg, Brambsa Christine E, Ortiza Javier U, Lobmaiera Silvia M (2017). Longitudinal progression of fetal short-term variation and average acceleration and deceleration capacity after antenatal maternal betamethasone application. European Journal of Obstetrics & Gynecology and Reproductive Biology.

[7] Jungers P (2004). Néphropathie et grossesse. EMC-Medecine.

